# Effect of Li Termination on the Electronic and Hydrogen Storage Properties of Linear Carbon Chains: A TAO-DFT Study

**DOI:** 10.1038/s41598-017-05202-6

**Published:** 2017-07-10

**Authors:** Sonai Seenithurai, Jeng-Da Chai

**Affiliations:** 1 Department of Physics, National Taiwan University, Taipei, 10617 Taiwan; 2 Center for Theoretical Sciences and Center for Quantum Science and Engineering, National Taiwan University, Taipei, 10617 Taiwan

## Abstract

Accurate prediction of the electronic and hydrogen storage properties of linear carbon chains (C_*n*_) and Li-terminated linear carbon chains (Li_2_C_*n*_), with *n* carbon atoms (*n* = 5–10), has been very challenging for traditional electronic structure methods, due to the presence of strong static correlation effects. To meet the challenge, we study these properties using our newly developed thermally-assisted-occupation density functional theory (TAO-DFT), a very efficient electronic structure method for the study of large systems with strong static correlation effects. Owing to the alteration of the reactivity of C_*n*_ and Li_2_C_*n*_ with *n*, odd-even oscillations in their electronic properties are found. In contrast to C_*n*_, the binding energies of H_2_ molecules on Li_2_C_*n*_ are in (or close to) the ideal binding energy range (about 20 to 40 kJ/mol per H_2_). In addition, the H_2_ gravimetric storage capacities of Li_2_C_*n*_ are in the range of 10.7 to 17.9 wt%, satisfying the United States Department of Energy (USDOE) ultimate target of 7.5 wt%. On the basis of our results, Li_2_C_*n*_ can be high-capacity hydrogen storage materials that can uptake and release hydrogen at temperatures well above the easily achieved temperature of liquid nitrogen.

## Introduction

Hydrogen (H_2_), as a pure energy carrier, has many attributes. Being lightweight, it carries 142 MJ/kg of energy, which is approximately three times the energy content of gasoline, in terms of mass. Also, it is highly abundant on the earth in the form of water. More importantly, when hydrogen is burned with oxygen, it releases water vapor as the only effluent. Despite these advantages, there remain several problems to be clarified for the use of hydrogen. For example, hydrogen is highly flammable, and hence, if it comes in contact with the environment, it will burst. Another problem is related to its low energy content in terms of volume: it has only 0.0180 MJ/L, which is very low relative to gasoline (34.8 MJ/L). Moreover, over the past few years, the storage of hydrogen for onboard applications has been an active arena, which also requires a lightweight storage medium. Because of these reasons, storing a large amount of hydrogen reversibly in a small and lightweight container safely has been the biggest challenge in realizing a hydrogen-based economy^1–5^.

Over the years, the United States Department of Energy (USDOE) has monitored the research progress in the development of hydrogen storage materials for consumer vehicles. In 2015, the USDOE set the ultimate target of 7.5 wt% for the gravimetric storage capacities of onboard hydrogen storage materials for light-duty vehicles^5^. As of now, there have been several methods for the storage of hydrogen^1–4^. The conventional methods for storing hydrogen are the high pressure method and the cryogenic method. In the high pressure method, one adopts carbon fiber reinforced tanks, which can withstand very high pressures (e.g., 350 to 700 bar), to store a large amount of completely recoverable hydrogen. In the cryogenic method, hydrogen is stored at very low temperatures (e.g., 20 K), typically requiring an expensive liquid helium refrigeration system. Both of these methods are not suitable for onboard automobile applications, because of the associated risk, high cost, and heavy weight. The storage of hydrogen in a metal hydride seems to be a convincing solution, but the irreversibility, slow kinetics, and high desorption temperature associated with this method are the problems yet to be overcome. Another promising solution is the storage of hydrogen in high surface area materials (e.g., graphene, carbon nanotubes, and metal-organic frameworks) through the adsorption-based methods. As high surface area materials could adsorb large amounts of hydrogen, the corresponding H_2_ gravimetric storage capacities could be rather high. Nevertheless, these materials bind H_2_ molecules very weakly (i.e., mainly governed by van der Waals (vdW) interactions), and hence, they perform properly only at very low temperatures.

For reversible hydrogen adsorption and desorption at ambient conditions (298 K and 1 bar), in addition to other thermodynamic considerations, the ideal binding energies of H_2_ molecules on hydrogen storage materials should be in the range of about 20 to 40 kJ/mol per H_2_^6–8^. Consequently, various novel methods are being explored to increase the binding energies of H_2_ molecules on high surface area materials to the aforementioned ideal range for ambient storage applications. To increase the H_2_ adsorption binding energy, the surface of the adsorbent is generally modified with substitution doping, adatom adsorption, functionalization, etc.^2^. Among them, Li adsorption is especially attractive, because of its light weight with which a high gravimetric storage capacity could be easily achieved. Note also that Li-adsorbed carbon materials have been shown to possess relatively high gravimetric storage capacities with enhanced H_2_ adsorption binding energies^9–17^, through a charge-transfer induced polarization mechanism^2, 18–20^.

**Figure 1:**
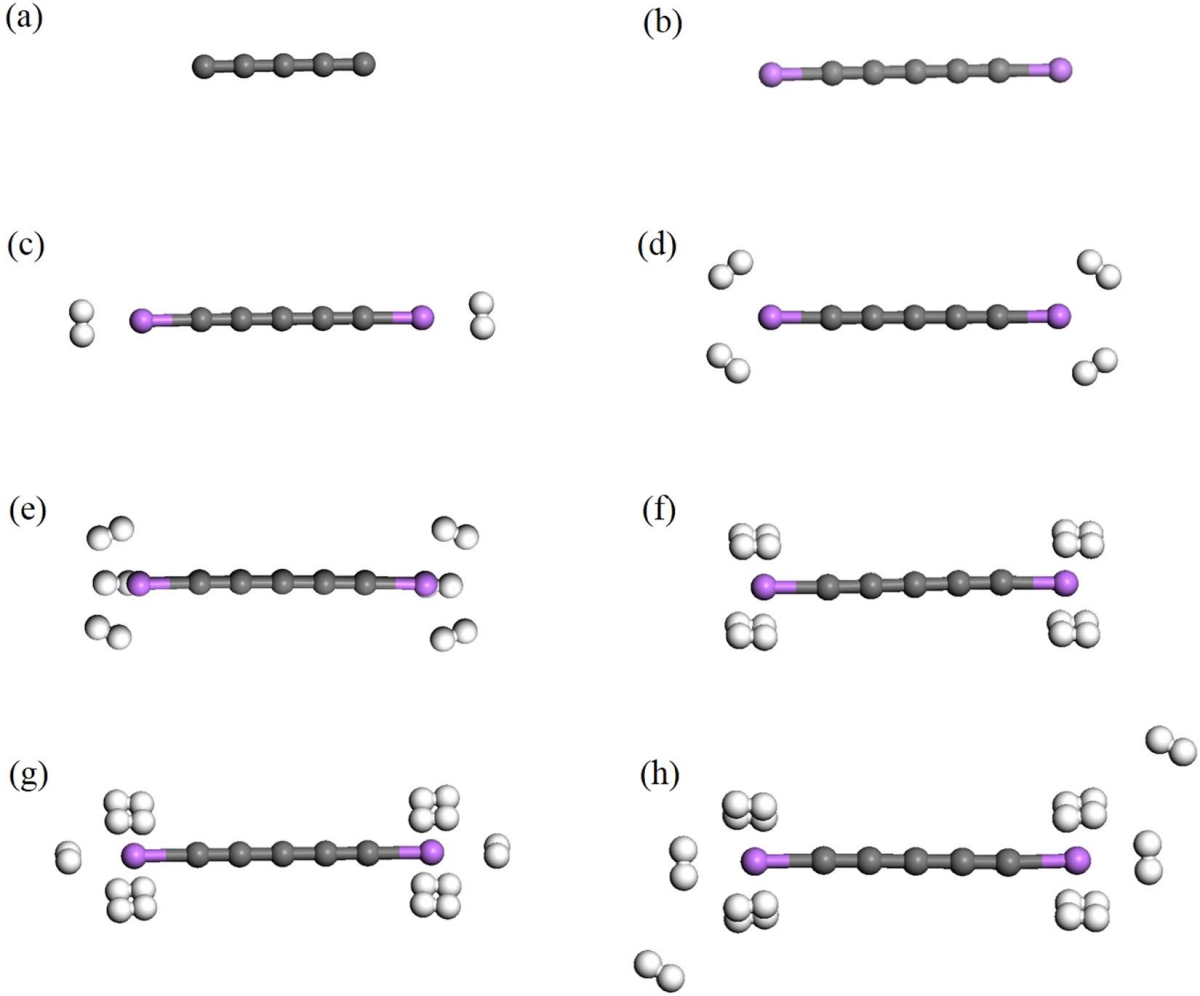
Structures of (**a**) C_5_, (**b**) Li_2_C_5_, (**c**) Li_2_C_5_ with one H_2_ molecule adsorbed on each Li atom, (**d**) Li_2_C_5_ with two H_2_ molecules adsorbed on each Li atom, (**e**) Li_2_C_5_ with three H_2_ molecules adsorbed on each Li atom, (**f**) Li_2_C_5_ with four H_2_ molecules adsorbed on each Li atom, (**g**) Li_2_C_5_ with five H_2_ molecules adsorbed on each Li atom, and (**h**) Li_2_C_5_ with six H_2_ molecules adsorbed on each Li atom. Here, grey, white, and purple balls represent C, H, and Li atoms, respectively.

Among carbon materials, linear carbon chains (C_*n*_), consisting of *n* carbon atoms bonded with sp^1^ hybridization (see Fig. 1(a), have recently attracted much attention owing to their unique electronic properties^21–35^. Note that C_*n*_ may be considered for hydrogen storage applications due to their one-dimensional (1D) structures and the feasibility of synthesis of C_*n*_ and their derivatives^24–30^. Recently, Pt-terminated linear carbon chains have been synthesized^28^. As mentioned above, due to a charge-transfer induced polarization mechanism^2, 18–20^, Li-terminated linear carbon chains (Li_2_C_*n*_) can be good candidates for hydrogen storage materials (see Fig. 1(b–h). Because of the light elements (i.e., C and Li atoms) in Li_2_C_*n*_, high gravimetric storage capacities could be easily achieved. However, to the best of our knowledge, there has been no comprehensive study on the electronic and hydrogen storage properties of Li_2_C_*n*_ in the literature, possibly due to the presence of strong static correlation effects in Li_2_C_*n*_ (commonly occurring in 1D structures due to quantum confinement effects)^36^. Theoretically, the popular Kohn-Sham density functional theory (KS-DFT)^37^ with conventional semilocal^38^, hybrid^39–42^, and double-hybrid^43–46^ exchange-correlation (XC) density functionals can provide unreliable results for systems with strong static correlation effects^47^. For the accurate prediction of the properties of these systems, high-level *ab initio* multi-reference methods are typically needed^48^. Nonetheless, accurate multi-reference calculations are prohibitively expensive for large systems (especially for geometry optimization).

To circumvent the formidable computational expense of high-level *ab initio* multi-reference methods, we have newly developed thermally-assisted-occupation density functional theory (TAO-DFT)^49–51^ for the study of large ground-state systems (e.g., containing up to a few thousand electrons) with strong static correlation effects. In contrast to KS-DFT, TAO-DFT is a density functional theory with fractional orbital occupations, wherein strong static correlation is explicitly described by the entropy contribution (see Eq. (26) of ref. 49), a function of the fictitious temperature and orbital occupation numbers. Note that the entropy contribution is completely missing in KS-DFT. Interestingly, TAO-DFT is as efficient as KS-DFT for single-point energy and analytical nuclear gradient calculations, and is reduced to KS-DFT in the absence of strong static correlation effects. Therefore, TAO-DFT can treat both single- and multi-reference systems in a more balanced way than KS-DFT. Besides, existing XC density functionals in KS-DFT may also be adopted in TAO-DFT. Due to its computational efficiency and reasonable accuracy for large systems with strong static correlation, TAO-DFT has been successfully applied to the study of several strongly correlated electron systems at the nanoscale^17, 52–54^, which are typically regarded as “challenging systems” for traditional electronic structure methods (e.g., KS-DFT with conventional XC density functionals and single-reference *ab initio* methods)^47^. Accordingly, TAO-DFT can be an ideal theoretical method for studying the electronic properties of Li_2_C_*n*_. Besides, the orbital occupation numbers in TAO-DFT can be useful for examining the possible radical character of Li_2_C_*n*_. For the hydrogen storage properties, as the interaction between H_2_ and Li_2_C_*n*_ may involve dispersion (vdW) interactions, electrostatic interactions, and orbital interactions^3, 7, 55^, the inclusion of dispersion corrections^56, 57^ in TAO-DFT is important for properly describing noncovalent interactions. Therefore, in this work, we adopt TAO-DFT with dispersion corrections^50^ to study the electronic and hydrogen storage properties of Li_2_C_*n*_ with various chain lengths (*n* = 5–10). In addition, the electronic properties of Li_2_C_*n*_ are also compared with those of C_*n*_ to examine the role of Li termination.

## Computational Details

All calculations are performed with a development version of Q-Chem 4.4^58^, using the 6–31G(d) basis set with the fine grid EML(75,302), consisting of 75 Euler-Maclaurin radial grid points and 302 Lebedev angular grid points. Results are computed using TAO-BLYP-D^50^ (i.e., TAO-DFT with the dispersion-corrected BLYP-D XC density functional^56^ and the LDA *θ*-dependent density functional ${E}_{\theta }^{{\rm{LDA}}}$ (see Eq. (41) of ref. 49) with the fictitious temperature *θ* = 7 mhartree (as defined in ref. 49).

## Results and Discussion

### Electronic Properties

**Figure 2:**
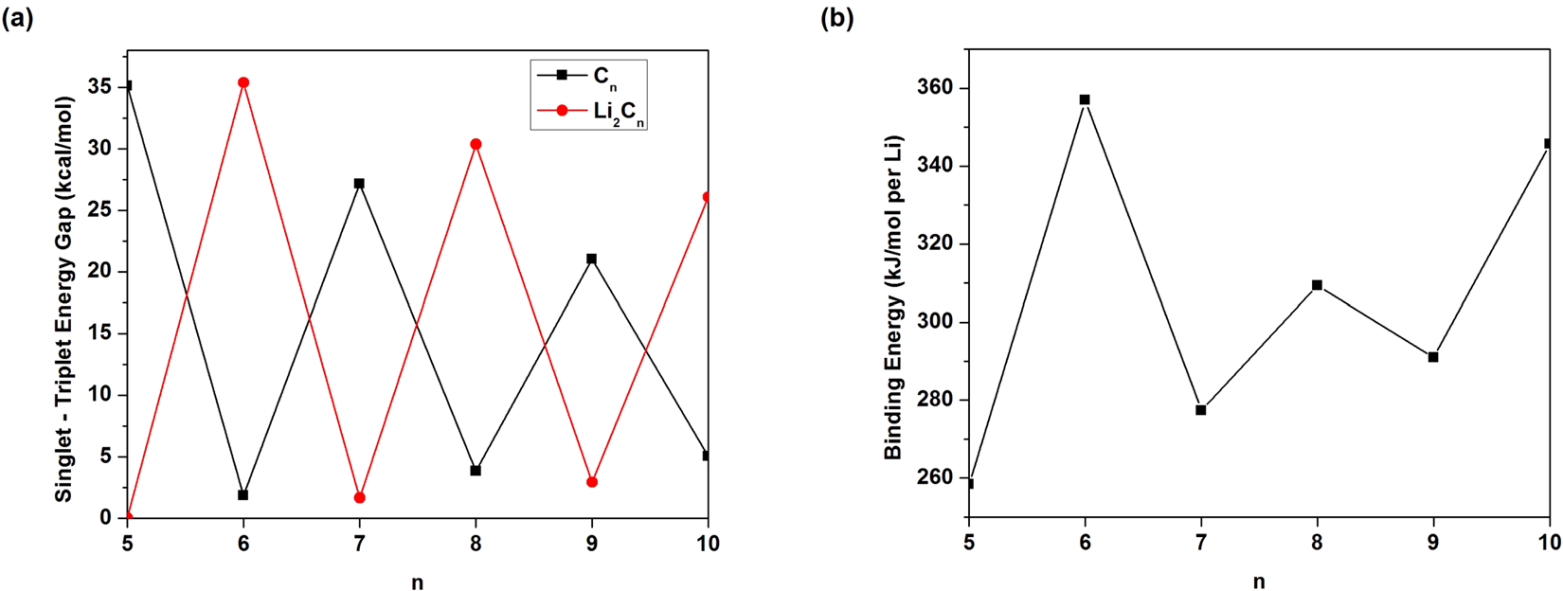
(**a**) Singlet-triplet energy (ST) gap of C_*n*_/Li_2_C_*n*_ and (**b**) Li binding energy on C_*n*_ as a function of the chain length, calculated using TAO-BLYP-D.

To obtain the ground state of C_*n*_/Li_2_C_*n*_ (*n* = 5–10), spin-unrestricted TAO-BLYP-D calculations are performed for the lowest singlet and triplet energies of C_*n*_/Li_2_C_*n*_ on the respective geometries that were fully optimized at the same level of theory. The singlet-triplet energy (ST) gap of C_*n*_/Li_2_C_*n*_ is calculated as (*E*_T_ − *E*_S_), the energy difference between the lowest triplet (T) and singlet (S) states of C_*n*_/Li_2_C_*n*_. As shown in Fig. 2(a), the ground states of C_*n*_ and Li_2_C_*n*_ are singlets for all the chain lengths investigated.

Because of the symmetry constraint, the spin-restricted and spin-unrestricted energies for the lowest singlet state of C_*n*_/Li_2_C_*n*_ should be the same for the exact theory^49–51, 59^. To assess the possible symmetry-breaking effects, we also perform spin-restricted TAO-BLYP-D calculations for the lowest singlet energies on the corresponding optimized geometries. The spin-restricted and spin-unrestricted TAO-BLYP-D energies for the lowest singlet state of C_*n*_/Li_2_C_*n*_ are found to be essentially the same (within the numerical accuracy of our calculations), implying that essentially no unphysical symmetry-breaking effects occur in our spin-unrestricted TAO-BLYP-D calculations.

To assess the energetic stability of terminating Li atoms, the Li binding energy, *E*_*b*_(Li), on C_*n*_ is computed using

1 $${E}_{b}({\rm{Li}})=({E}_{{{\rm{C}}}_{n}}+2{E}_{{\rm{Li}}}-{E}_{{{\rm{Li}}}_{2}{{\rm{C}}}_{n}}\mathrm{)/2,}$$

where ${E}_{{{\rm{C}}}_{n}}$ is the total energy of C_*n*_, *E*_Li_ is the total energy of Li, and ${E}_{{{\rm{Li}}}_{2}{{\rm{C}}}_{n}}$ is the total energy of Li_2_C_*n*_. *E*_*b*_(Li) is subsequently corrected for the basis set superposition error (BSSE) using the counterpoise correction^60^, where the C_*n*_ is considered as one fragment, and the 2 Li atoms are considered as the other fragment. As shown in Fig. 2(b), C_*n*_ can strongly bind the Li atoms with the binding energy range of 258 to 357 kJ/mol per Li.

**Figure 3:**
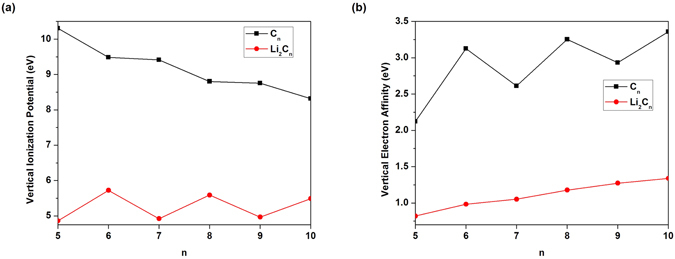
(**a**) Vertical ionization potential and (**b**) vertical electron affinity for the lowest singlet state of C_*n*_/Li_2_C_*n*_ as a function of the chain length, calculated using TAO-BLYP-D.

**Figure 4:**
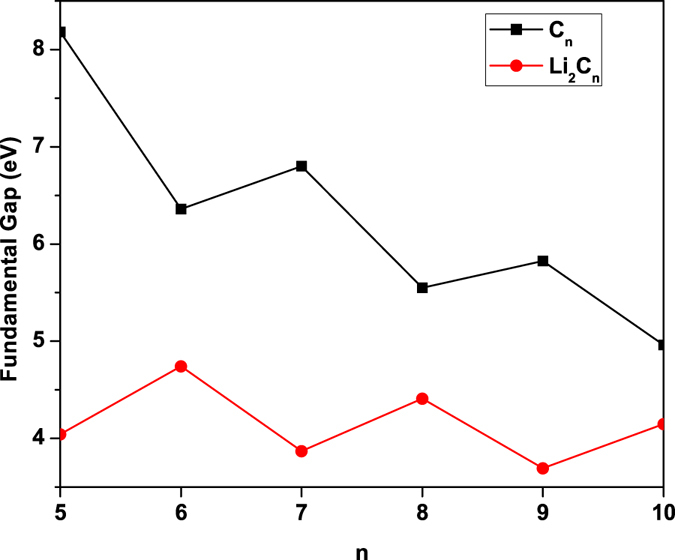
Fundamental gap for the lowest singlet state of C_*n*_/Li_2_C_*n*_ as a function of the chain length, calculated using TAO-BLYP-D.

At the ground-state (i.e., the lowest singlet state) geometry of C_*n*_/Li_2_C_*n*_ (with *N* electrons), the vertical ionization potential (IP_*v*_ = *E*_*N*−1_ − *E*_*N*_), vertical electron affinity (EA_*v*_ = *E*_*N*_ − *E*_*N*+1_), and fundamental gap (*E*_*g*_ = IP_*v*_ − EA_*v*_ = *E*_*N*+1_ + *E*_*N*−1_ − 2*E*_*N*_) are obtained with multiple energy-difference calculations, with *E*_*N*_ being the total energy of the *N*-electron system. For each *n*, Li_2_C_*n*_ possesses the smaller IP_*v*_ (see Fig. 3(a), EA_*v*_ (see Fig. 3(b), and *E*_*g*_ (see Fig. 4) values than C_*n*_. Note also that the IP_*v*_, EA_*v*_, and *E*_*g*_ values of Li_2_C_*n*_ are less sensitive to the chain length than those of C_*n*_.

**Figure 5:**
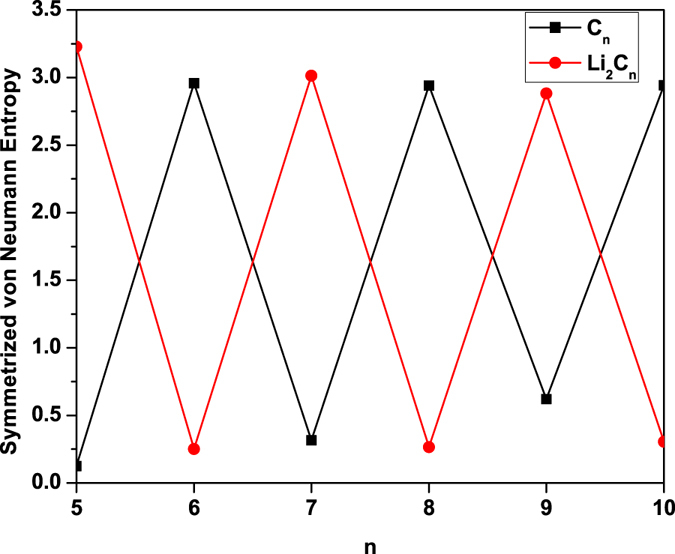
Symmetrized von Neumann entropy for the lowest singlet state of C_*n*_/Li_2_C_*n*_ as a function of the chain length, calculated using TAO-BLYP-D.

To examine the possible radical character of C_*n*_/Li_2_C_*n*_, we calculate the symmetrized von Neumann entropy (e.g., see Eq. (9) of ref. 59)

2 $${S}_{{\rm{vN}}}=-\frac{1}{2}\sum _{i=1}^{\infty }\{\,{f}_{i}\,\mathrm{ln}({f}_{i})+(1-{f}_{i})\,\mathrm{ln}(1-{f}_{i})\}$$

for the lowest singlet state of C_*n*_/Li_2_C_*n*_ as a function of the chain length, using TAO-BLYP-D. Here, *f*_*i*_ the occupation number of the *i* ^th^ orbital obtained with TAO-BLYP-D, which varies from 0 to 1, is approximately equal to the occupation number of the *i* ^th^ natural orbital^49–51, 61^. For a system without strong static correlation ({*f*_*i*_} are close to either 0 or 1), *S*_vN_ provides insignificant contributions, while for a system with strong static correlation ({*f*_*i*_} are fractional for active orbitals, and are close to either 0 or 1 for others), *S*_vN_ increases with the number of active orbitals. As shown in Fig. 5, the *S*_vN_ values of C_*n*_ with even-number carbon atoms and Li_2_C_*n*_ with odd-number carbon atoms are much larger than the *S*_vN_ values of C_*n*_ with odd-number carbon atoms and Li_2_C_*n*_ with even-number carbon atoms, respectively.

**Figure 6:**
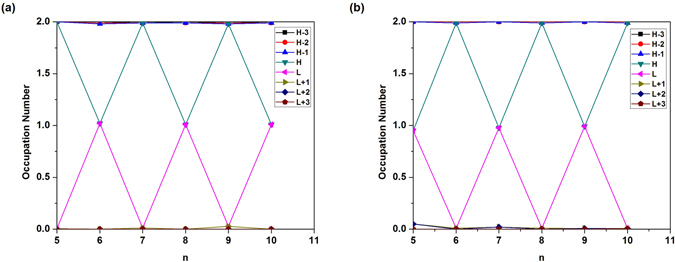
Occupation numbers of the active orbitals (HOMO − 3, HOMO − 2, HOMO − 1, HOMO, LUMO, LUMO + 1, LUMO + 2, and LUMO + 3) for the lowest singlet states of (**a**) C_*n*_ and (**b**) Li_2_C_*n*_, calculated using TAO-BLYP-D. For brevity, HOMO is denoted as H, LUMO is denoted as L, and so on.

To illustrate the causes of the odd-even oscillations in *S*_vN_, we plot the occupation numbers of the active orbitals for the lowest singlet states of C_*n*_ (see Fig. 6(a) and Li_2_C_*n*_ (see Fig. 6(b), calculated using TAO-BLYP-D. Here, the highest occupied molecular orbital (HOMO) is the (*N*/2)^th^ orbital, and the lowest unoccupied molecular orbital (LUMO) is the (*N*/2 + 1)^th^ orbital, with *N* being the number of electrons in C_*n*_/Li_2_C_*n*_. For brevity, HOMO, HOMO − 1, HOMO − 2, and HOMO − 3, are denoted as H, H − 1, H − 2, and H − 3, respectively, while LUMO, LUMO + 1, LUMO + 2, and LUMO + 3, are denoted as L, L + 1, L + 2, and L + 3, respectively. As shown, C_*n*_ with even-number carbon atoms and Li_2_C_*n*_ with odd-number carbon atoms possess more pronounced diradical character than C_*n*_ with odd-number carbon atoms and Li_2_C_*n*_ with even-number carbon atoms, respectively.

On the basis of several measures (e.g., the smaller ST gap, smaller *E*_*g*_, larger *S*_vN_, and more pronounced diradical character), C_*n*_ with even-number carbon atoms and Li_2_C_*n*_ with odd-number carbon atoms should exhibit much stronger static correlation effects than C_*n*_ with odd-number carbon atoms and Li_2_C_*n*_ with even-number carbon atoms (i.e., possessing single-reference character), respectively. Note that KS-DFT with conventional XC density functionals can be unreliable for the properties of systems with strong static correlation effects, and accurate multi-reference calculations are prohibitively expensive for large systems (e.g., the longer C_*n*_ and Li_2_C_*n*_). In addition, due to the alteration of the reactivity of C_*n*_ and Li_2_C_*n*_ with *n*, it is highly desirable to adopt an electronic structure method that can provide a balanced performance for both single- and multi-reference systems, well justifying the use of TAO-DFT in this study.

### Hydrogen Storage Properties

As pure carbon materials bind H_2_ molecules very weakly (i.e., mainly governed by vdW interactions), they are unlikely to be promising hydrogen storage materials at ambient conditions^6^. Similarly, C_*n*_ are not ideal for ambient storage applications, since the binding energies of H_2_ molecules remain small. In addition, the number of H_2_ molecules that can be adsorbed on C_*n*_ is quite limited, due to the repulsive interaction between the adsorbed H_2_ molecules at short distances^62^. Consequently, the more the adsorbed H_2_ molecules, the less the average H_2_ binding energy on C_*n*_. Therefore, C_*n*_ cannot be high-capacity hydrogen storage materials at ambient conditions.

**Figure 7:**
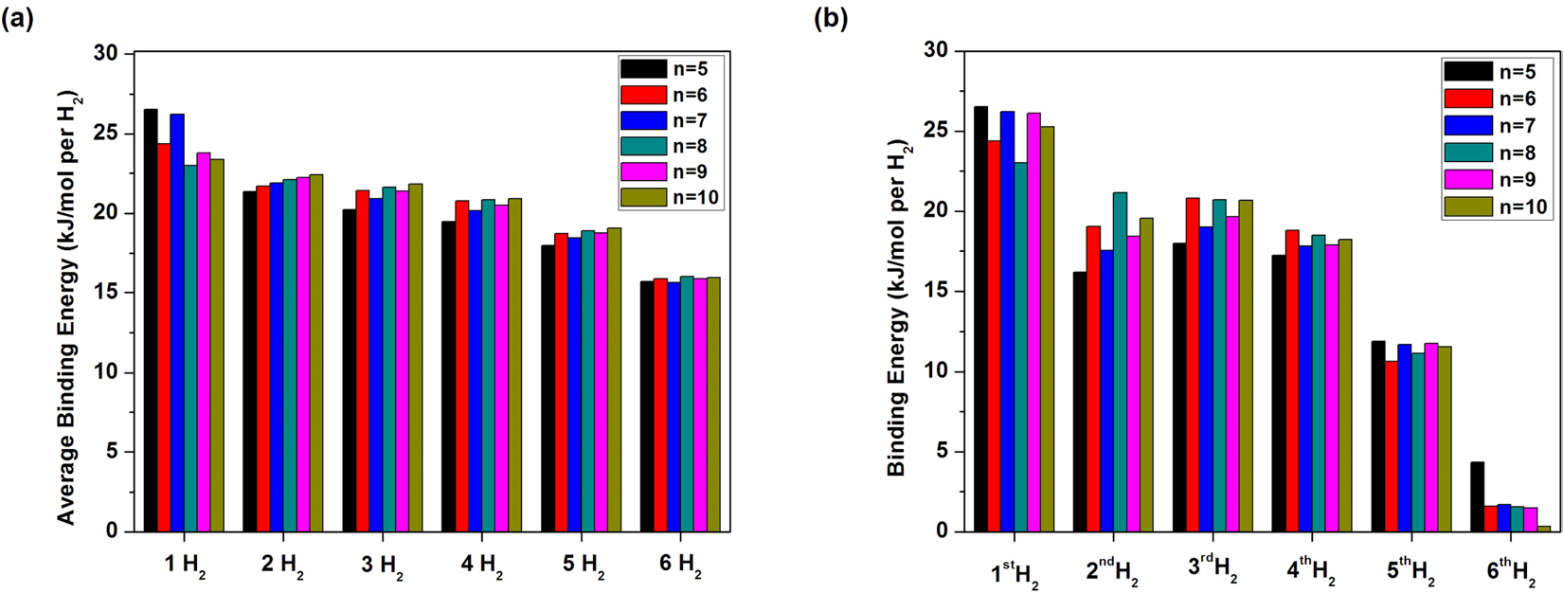
(**a**) Average H_2_ binding energy on Li_2_C_*n*_ (*n* = 5–10) as a function of the number of H_2_ molecules adsorbed on each Li atom, and (**b**) the binding energy of the *y* ^th^ H_2_ molecule (*y* = 1–6) on Li_2_C_*n*_ (*n* = 5–10), calculated using TAO-BLYP-D.

Here, we investigate the hydrogen storage properties of Li_2_C_*n*_ (*n* = 5–10). As illustrated in Fig. 1(b–h), at the ground-state geometry of Li_2_C_*n*_, *x* H_2_ molecules (*x* = 1–6) are initially placed on various possible sites around each Li atom, and the structures are subsequently optimized to obtain the most stable geometry. All the H_2_ molecules are found to be adsorbed molecularly to the Li atoms. The average H_2_ binding energy, *E*_*b*_(H_2_), on Li_2_C_*n*_ is evaluated by

3 $${E}_{b}({{\rm{H}}}_{2})=({E}_{{{\rm{Li}}}_{2}{{\rm{C}}}_{n}}+2x{E}_{{{\rm{H}}}_{2}}-{E}_{{{\rm{Li}}}_{2}{{\rm{C}}}_{n}-2x{{\rm{H}}}_{2}}\mathrm{)/(2}x),$$

where ${E}_{{{\rm{H}}}_{2}}$ is the total energy of H_2_, and ${E}_{{{\rm{Li}}}_{2}{{\rm{C}}}_{n}-2x{{\rm{H}}}_{2}}$ is the total energy of Li_2_C_*n*_ with *x* H_2_ molecules adsorbed on each Li atom. Subsequently, *E*_*b*_(H_2_) is corrected for BSSE using a standard counterpoise correction^60^. As shown in Fig. 7(a), *E*_*b*_(H_2_) is in the range of 19 to 27 kJ/mol per H_2_ for *x* = 1–4, in the range of 18 to 19 kJ/mol per H_2_ for *x* = 5, and about 16 kJ/mol per H_2_ for *x* = 6, falling in (or close to) the ideal binding energy range.

To assess if the binding energies of successive H_2_ molecules are also in (or close to) the ideal binding energy range (i.e., not just the average H_2_ binding energy), the binding energy of the *y* ^th^ H_2_ molecule (*y* = 1–6), *E*_*b*,*y*_(H_2_), on Li_2_C_*n*_ is evaluated by

4 $${E}_{b,y}({{\rm{H}}}_{2})=({E}_{{{\rm{Li}}}_{2}{{\rm{C}}}_{n}-\mathrm{2(}y-\mathrm{1)}{{\rm{H}}}_{2}}+2{E}_{{{\rm{H}}}_{2}}-{E}_{{{\rm{Li}}}_{2}{{\rm{C}}}_{n}-2y{{\rm{H}}}_{2}}\mathrm{)/2.}$$

Similarly, *E*_*b*,*y*_(H_2_) is also corrected for BSSE using a standard counterpoise correction^60^. As shown in Fig. 7(b), *E*_*b*,*y*_(H_2_) is in the range of 16 to 27 kJ/mol per H_2_ for *y* = 1–4, in the range of 11 to 12 kJ/mol per H_2_ for *y* = 5, and less than 5 kJ/mol per H_2_ for *y* = 6. Therefore, while the first four H_2_ molecules can be adsorbed on Li_2_C_*n*_ in (or close to) the ideal binding energy range, the fifth and sixth H_2_ molecules are only weakly adsorbed (i.e., appropriate only for storage at very low temperatures).

**Figure 8:**
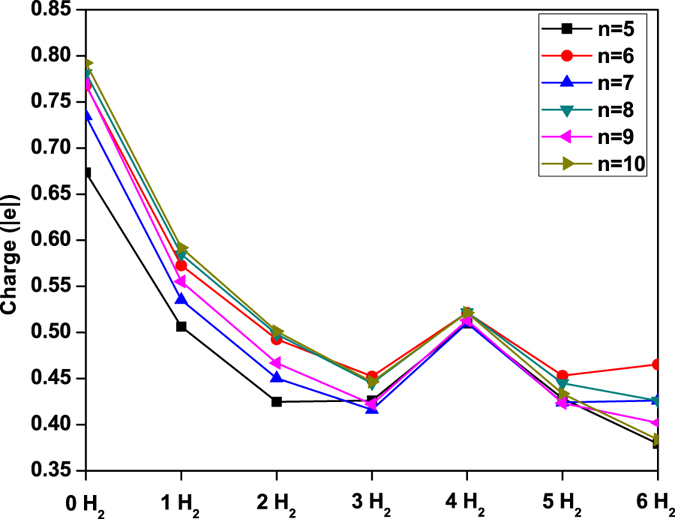
CHELPG atomic charge on each Li atom for Li_2_C_*n*_ (*n* = 5–10) with *x* H_2_ molecules (*x* = 0–6) adsorbed on each Li atom, calculated using TAO-BLYP-D.

**Figure 9:**
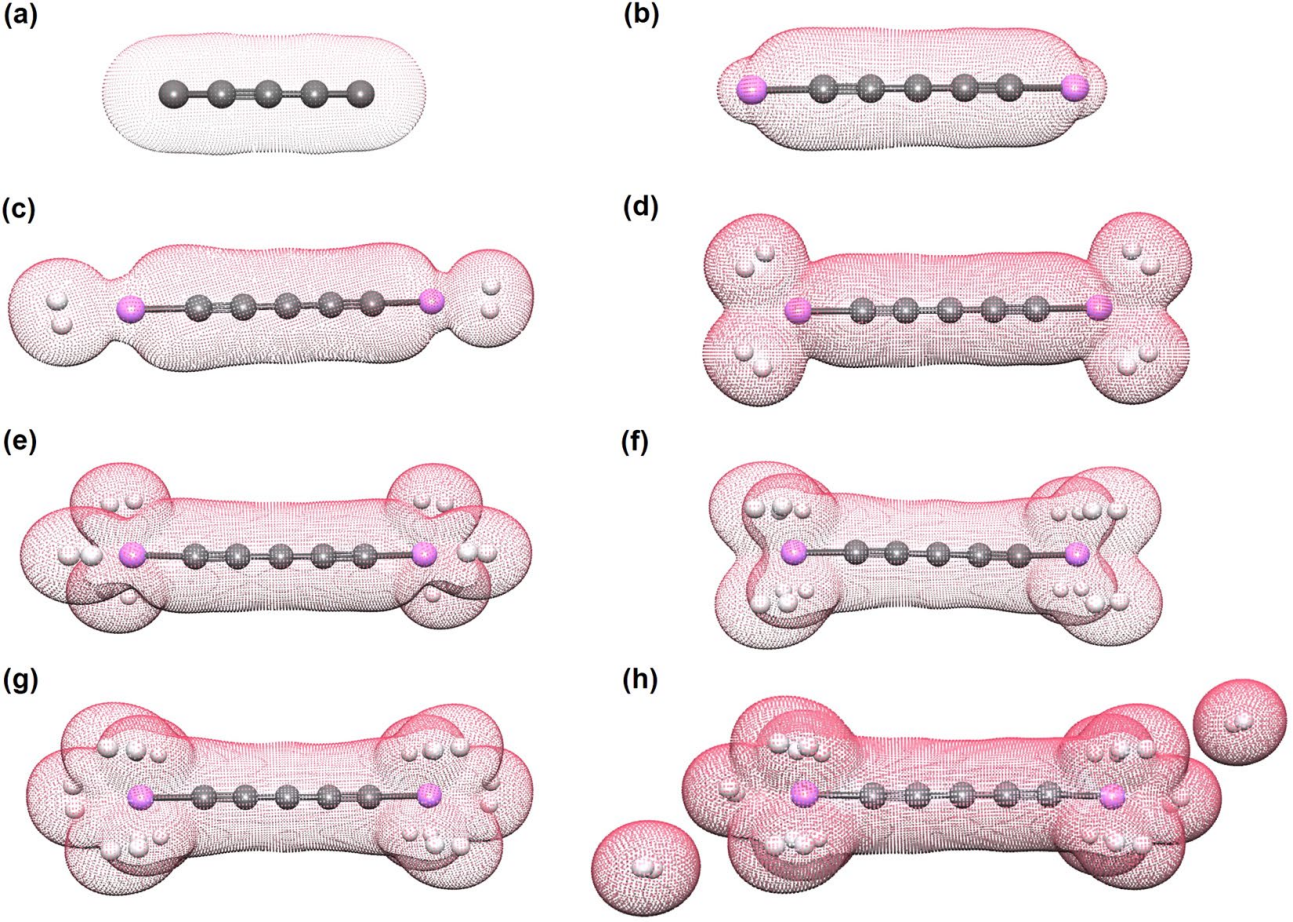
Charge density isosurfaces of (**a**) C_5_ and (**b**–**h**) Li_2_C_5_ with *x* H_2_ molecules (*x* = 0–6) adsorbed on each Li atom, calculated using TAO-BLYP-D, at isovalue = 0.02 e/Å^3^. Here, grey, white, and purple balls represent C, H, and Li atoms, respectively.

To assess the types of noncovalent interactions between H_2_ and Li_2_C_*n*_, we compute the atomic charge on each Li atom for Li_2_C_*n*_ (*n* = 5–10) with *x* H_2_ molecules (*x* = 0–6) adsorbed on each Li atom (see Fig. 8), using the CHELPG (CHarges from ELectrostatic Potentials using a Grid based method) scheme^63^, in which atomic charges are fitted to reproduce the molecular electrostatic potential at a number of points around the molecule. For further clarification, we also plot the charge density isosurfaces of C_5_ and Li_2_C_5_ with *x* H_2_ molecules (*x* = 0–6) adsorbed on each Li atom (see Fig. 9). Similar charge density isosurfaces are also found for the longer Li_2_C_*n*_ (*n* = 6–10) with the same number of adsorbed H_2_ molecules. As the electronegativity of C is much higher than that of Li, the transfer of electronic charge in Li_2_C_*n*_ is from Li to C_*n*_, resulting in a positive charge of 0.67–0.79 |*e*| on each Li atom in Li_2_C_*n*_. The positively charged Li atom can interact with more than one H_2_ molecule, but the positive charge on Li decreases for the subsequent adsorption of H_2_ molecules (up to *x* = 3). This type of adsorption can be attributed to the polarization of H_2_ molecules by the positively charged Li atom (i.e., charge-induced dipole interaction)^2, 18–20^, leading to the enhanced H_2_ binding energy and high hydrogen uptake in Li_2_C_*n*_. When the number of adsorbed H_2_ molecules is large (e.g., *x* = 4–6), there is a significant overlap of the Li and H_2_ charge densities, enhancing orbital interactions^3, 7, 55^. This suggests that orbital interactions should also be responsible for the H_2_ binding energy, especially when a large number of H_2_ molecules (e.g., *x* = 4–6) are adsorbed on the Li atom. In particular, due to the enhanced orbital interactions, when the fourth H_2_ molecule is adsorbed on the Li atom, a small fraction of electronic charge is transferred from the Li atom to the adsorbed H_2_ molecules, slightly increasing the positive charge on Li. Interestingly, there is no overlap between the charge density of the sixth H_2_ molecule and the charge densities of other molecules, supporting that the sixth H_2_ molecule is only weakly adsorbed (i.e., mainly governed by vdW interactions). Accordingly, the noncovalent interactions between H_2_ and Li_2_C_*n*_ should involve charge-induced dipole interactions, orbital interactions, and vdW interactions.

The desorption temperature, *T*_*D*_, of the adsorbed H_2_ molecules is estimated using the van’t Hoff equation^13, 17, 64, 65^,

5 $${T}_{D}=\frac{{E}_{b}({{\rm{H}}}_{2})}{{k}_{B}}{\{\frac{{\rm{\Delta }}S}{R}-\mathrm{ln}\frac{{p}_{0}}{{p}_{eq}}\}}^{-1},$$

where *E*_*b*_(H_2_) is the average H_2_ binding energy (given by Eq. (3)), Δ*S* is the change in hydrogen entropy from gas to liquid phase (Δ*S* = 13.819*R* taken from ref. 66), *p*_0_ is the standard atmospheric pressure (1 bar), *p*_*eq*_ is the equilibrium pressure, *k*_*B*_ is the Boltzmann constant, and *R* is the gas constant. As shown in Table 1, *T*_*D*_ for Li_2_C_*n*_ (*n* = 5–10) with *x* H_2_ molecules (*x* = 1–4) adsorbed on each Li atom, is estimated using Eq. (5) at *p*_*eq*_ = 1.5 bar (as adopted in ref. 6) and at *p*_*eq*_ = 1 bar (the standard atmospheric pressure). As the *E*_*b*_(H_2_) values are in the range of 19.47 to 26.53 kJ/mol per H_2_ for *x* = 1–4, the corresponding *T*_*D*_ values are in the range of 165 to 224 K at *p*_*eq*_ = 1.5 bar, and in the range of 169 to 231 K at *p*_*eq*_ = 1 bar, well above the easily achieved temperature of liquid nitrogen (i.e., 77 K). Therefore, Li_2_C_*n*_ (*n* = 5–10) can be viable hydrogen storage materials that can uptake and release hydrogen at temperatures well above 77 K. Note that strictly, Δ*S* should be the change of total entropy before and after the hydrogenation. Therefore, the *T*_*D*_ values given in Table 1 have to be taken as rough estimates for the desorption temperatures due to the definition of Δ*S*. For all metal-hydrogen systems, Δ*S* can be roughly estimated as the entropy change from molecular hydrogen gas to dissolved solid hydrogen^1^, that is 15.720*R* (taken from ref. 66), as it arises mainly from the entropy loss of gaseous hydrogen during hydrogen uptake by the metal. Since Li_2_C_*n*_ (*n* = 5–10) adsorb hydrogen as a form of molecule, Δ*S* should be smaller than 15.720*R* (as the entropy of adsorbed hydrogen should be positive yet nonvanishingly small). While there is no accurate estimate of the entropy of hydrogen in adsorbed state, it should be safe to assume that it is much less than that of the gas state^67^. Therefore, we estimate Δ*S* as the entropy change from molecular hydrogen gas to liquid hydrogen, as suggested by previous studies^13, 17, 64, 65^. On the basis of Eq. (5), the larger the Δ*S*, the lower the *T*_*D*_ values. However, even if the maximal Δ*S* (i.e., 15.720*R*) is adopted, the corresponding *T*_*D*_ values will be only slightly lower (i.e., within 28 K for each case) than our reported *T*_*D*_ values given in Table 1, being also well above 77 K. Accordingly, our comments remain the same even for the extreme case.

**Table 1:** Average H_2_ binding energy *E*_*b*_(H_2_) (kJ/mol per H_2_) and H_2_ desorption temperature *T*_*D*_ (K) for Li_2_C_*n*_ (*n* = 5–10) with *x* H_2_ molecules (*x* = 1–4) adsorbed on each Li atom, calculated using TAO-BLYP-D.

*n*	*E*_*b*_(H_2_)				*T*_*D*_ (*p*_*eq*_ = 1.5)				*T*_*D*_ (*p*_*eq*_ = 1)			
	1 H_2_	2 H_2_	3 H_2_	4 H_2_	1 H_2_	2 H_2_	3 H_2_	4 H_2_	1 H_2_	2 H_2_	3 H_2_	4 H_2_
5	26.53	21.35	20.22	19.47	224	181	171	165	231	186	176	169
6	24.37	21.70	21.41	20.76	206	184	181	176	212	189	186	181
7	26.21	21.88	20.91	20.14	222	185	177	170	228	190	182	175
8	23.01	22.10	21.62	20.84	195	187	183	176	200	192	188	181
9	23.77	22.25	21.38	20.51	201	188	181	173	207	194	186	179
10	23.39	22.40	21.82	20.92	198	189	185	177	204	195	190	182

Here, *T*_*D*_ is estimated using the van’t Hoff equation (see Eq. (5)) at *p*_*eq*_ = 1.5 (bar) and at *p*_*eq*_ = 1 (bar).

As Li_2_C_*n*_ (*n* = 5–10) can bind up to 8 H_2_ molecules (i.e., each Li atom can bind up to 4 H_2_ molecules) with the average and successive H_2_ binding energies in (or close to) the ideal binding energy range, the corresponding H_2_ gravimetric storage capacity, *C*_*g*_, is calculated using

6 $${C}_{g}=\frac{8{M}_{{{\rm{H}}}_{2}}}{{M}_{{{\rm{Li}}}_{2}{{\rm{C}}}_{n}}+8{M}_{{{\rm{H}}}_{2}}}\mathrm{.}$$

Here, ${M}_{{{\rm{Li}}}_{2}{{\rm{C}}}_{n}}$ is the mass of Li_2_C_*n*_, and ${M}_{{{\rm{H}}}_{2}}$ is the mass of H_2_. Note that *C*_*g*_ (see Eq. (6)) is 17.9 wt% for *n* = 5, 15.8 wt% for *n* = 6, 14.1 wt% for *n* = 7, 12.8 wt% for *n* = 8, 11.7 wt% for *n* = 9, and 10.7 wt% for *n* = 10, satisfying the USDOE ultimate target of 7.5 wt%. Based on the observed trends for Li_2_C_*n*_, the maximum number of H_2_ molecules that can be adsorbed on each Li atom with the average and successive H_2_ binding energies in (or close to) the ideal binding energy range should be 4, regardless of the chain length. Therefore, the *C*_*g*_ value of Li_2_C_*n*_ should decrease as the chain length increases. Note, however, that the *C*_*g*_ values obtained here may not be directly compared to the USDOE target value, which refers to the complete storage system (i.e., with the storage material, enclosing tank, insulation, piping, etc.)^5^. Nevertheless, since the *C*_*g*_ values obtained here are much higher (especially for the shorter Li_2_C_*n*_) than the USDOE ultimate target, the complete storage systems based on Li_2_C_*n*_ are likely to be high-capacity hydrogen storage materials that can uptake and release hydrogen at temperatures well above the temperature of liquid nitrogen.

## Conclusions

In conclusion, the search for ideal hydrogen storage materials has been extended to large systems with strong static correlation effects (i.e., those beyond the reach of traditional electronic structure methods), due to recent advances in TAO-DFT. In this work, we have studied the electronic properties (i.e., the Li binding energies, ST gaps, vertical ionization potentials, vertical electron affinities, fundamental gaps, symmetrized von Neumann entropy, and active orbital occupation numbers) and hydrogen storage properties (i.e., the average H_2_ binding energies, successive H_2_ binding energies, H_2_ desorption temperatures, and H_2_ gravimetric storage capacities) of Li_2_C_*n*_ (*n* = 5–10) using TAO-DFT. As Li_2_C_*n*_ with odd-number carbon atoms have been shown to possess pronounced diradical character, KS-DFT with conventional XC density functionals can be unreliable for studying the properties of these systems. In addition, accurate multi-reference calculations are prohibitively expensive for the longer Li_2_C_*n*_ (especially for geometry optimization), and hence, the use of TAO-DFT in this study is well justified. On the basis of our results, Li_2_C_*n*_ can bind up to 8 H_2_ molecules (i.e., each Li atom can bind up to 4 H_2_ molecules) with the average and successive H_2_ binding energies in (or close to) the ideal range of about 20 to 40 kJ/mol per H_2_. Accordingly, the H_2_ gravimetric storage capacities of Li_2_C_*n*_ are in the range of 10.7 to 17.9 wt%, satisfying the USDOE ultimate target of 7.5 wt%. Consequently, Li_2_C_*n*_ can be high-capacity hydrogen storage materials that can uptake and release hydrogen at temperatures well above the easily achieved temperature of liquid nitrogen.

For the practical realization of hydrogen storage based on Li_2_C_*n*_, Li_2_C_*n*_ may be adopted as building blocks. For example, we may follow the proposal of Liu *et al*.^68^, and consider connecting Li-coated fullerenes with Li_2_C_*n*_, which could also serve as high-capacity hydrogen storage materials. A systematic study of the electronic and hydrogen storage properties of these systems is essential, and may be considered for future work. Since linear carbon chains^26, 27^ and Pt-terminated linear carbon chains^28^ have been successfully synthesized, the realization of hydrogen storage materials based on Li_2_C_*n*_ should be feasible, and is now open to experimentalists. In the future, we plan to examine how the electronic and hydrogen storage properties of linear carbon chains vary with different metal dopants (e.g., Na, Al, Ca, Ti, etc.).

## References

[1] Schlapbach L, Züttel A (2001). Hydrogen-storage materials for mobile applications. Nature.

[2] Jena P (2011). Materials for hydrogen storage: past, present, and future. J. Phys. Chem. Lett..

[3] Park N (2012). Progress on first-principles-based materials design for hydrogen storage. PNAS.

[4] Dalebrook AF, Gan W, Grasemann M, Moret S, Laurenczy G (2013). Hydrogen storage: beyond conventional methods. Chem. Commun..

[5] U. S. Department of Energy, *Target explanation document: onboard hydrogen storage for light-duty fuel cell vehicles. Technical report*. Available at: https://energy.gov/eere/fuelcells/hydrogen-storage (Accessed: January 2017) (2015).

[6] Bhatia SK, Myers AL (2006). Optimum conditions for adsorptive storage. Langmuir.

[7] Lochan RC, Head-Gordon M (2006). Computational studies of molecular hydrogen binding affinities: the role of dispersion forces, electrostatics, and orbital interactions. Phys. Chem. Chem. Phys..

[8] Sumida K (2013). Impact of metal and anion substitutions on the hydrogen storage properties of M-BTT metal-organic frameworks. J. Am. Chem. Soc..

[9] Chen P, Wu X, Lin J, Tan KL (1999). High H_2_ uptake by alkali-doped carbon nanotubes under ambient pressure and moderate temperatures. Science.

[10] Deng W-Q, Xu X, Goddard WA (2004). New alkali doped pillared carbon materials designed to achieve practical reversible hydrogen storage for transportation. Phys. Rev. Lett..

[11] Li A (2010). Lithium-doped conjugated microporous polymers for reversible hydrogen storage. Angew. Chemie Int. Ed..

[12] Seenithurai S, Kodi Pandyan R, Vinodh Kumar S, Saranya C, Mahendran M (2014). Li-decorated double vacancy graphene for hydrogen storage application: a first principles study. Int. J. Hydrogen Energy.

[13] Qiu N-X, Zhang C-H, Xue Y (2014). Tuning hydrogen storage in lithium-functionalized BC_2_N sheets by doping with boron and carbon. Chem Phys Chem.

[14] Hussain T, De Sarkar A, Ahuja R (2014). Functionalization of hydrogenated graphene by polylithiated species for efficient hydrogen storage. Int. J. Hydrogen Energy.

[15] Hussain T (2015). Hydrogen storage properties of light metal adatoms (Li, Na) decorated fluorographene monolayer. Nanotechnology.

[16] Hussain T, Hankel M, Searles DJ (2016). Computational evaluation of lithium-functionalized carbon nitride (g-C6N8) monolayer as an efficient hydrogen storage material. J. Phys. Chem. C.

[17] Seenithurai S, Chai J-D (2016). Effect of Li adsorption on the electronic and hydrogen storage properties of acenes: a dispersion-corrected TAO-DFT study. Sci. Rep..

[18] Niu J, Rao BK, Jena P (1992). Binding of hydrogen molecules by a transition-metal ion. Phys. Rev. Lett..

[19] Niu J, Rao BK, Jena P, Manninen M (1995). Interaction of H_2_ and He with metal atoms, clusters, and ions. Phys. Rev. B.

[20] Froudakis GE (2001). Why alkali-metal-doped carbon nanotubes possess high hydrogen uptake. Nano Lett..

[21] Fan Q, Pfeiffer GV (1989). Theoretical study of linear C_*n*_ (*n* = 6–10) and HC_*n*_H (*n* = 2–10) molecules. Chem. Phys. Lett..

[22] Heimann, R. B. In *Carbyne and Carbynoid Structures* (eds Heimann, R. B. *et al*.) (Kluwer Academic Publishers, 1999).

[23] Horný L, Petraco NDK, Schaefer HF (2002). Odd carbon long linear chains HC_2*n*+1_H (*n* = 4–11): Properties of the neutrals and radical anions. J. Am. Chem. Soc..

[24] Van Zee RJ, Ferrante RF, Zeringue KJ, Weltner W, Ewing DW (1988). Electron spin resonance of the C_6_, C_8_, and C_10_ molecules. J. Chem. Phys..

[25] Pan L, Rao BK, Gupta AK, Das GP, Ayyub P (2003). H-substituted anionic carbon clusters C_*n*_H^−^ (n ≤ 10): Density functional studies and experimental observations. J. Chem. Phys..

[26] Jin C, Lan H, Peng L, Suenaga K, Iijima S (2009). Deriving carbon atomic chains from graphene. Phys. Rev. Lett..

[27] Chuvilin A, Meyer JC, Algara-Siller G, Kaiser U (2009). From graphene constrictions to single carbon chains. New J. Phys..

[28] Kano E, Takeguchi M, Fujita J-I, Hashimoto A (2014). Direct observation of Pt-terminating carbyne on graphene. Carbon.

[29] Banhart F (2015). Chains of carbon atoms: a vision or a new nanomaterial?. Beilstein J. Nanotechnol..

[30] Casari CS, Tommasini M, Tykwinski RR, Milani A (2016). Carbon-atom wires: 1-D systems with tunable properties. Nanoscale.

[31] Belau L (2007). Ionization thresholds of small carbon clusters: tunable VUV experiments and theory. J. Am. Chem. Soc..

[32] Lang ND, Avouris P (1998). Oscillatory conductance of carbon-atom wires. Phys. Rev. Lett..

[33] Souza AMC, Herrmann H (2008). Theory of local electronic properties and finite-size effects in nanoscale open chains. Phys. Rev. B.

[34] Li ZY (2009). Magnetism and spin-polarized transport in carbon atomic wires. Phys. Rev. B.

[35] Artyukhov VI, Liu M, Yakobson BI (2014). Mechanically induced metal-insulator transition in carbyne. Nano Lett..

[36] Brus L (2014). Size, dimensionality, and strong electron correlation in nanoscience. Acc. Chem. Res..

[37] Kohn W, Sham LJ (1965). Self-consistent equations including exchange and correlation effects. Phys. Rev..

[38] Perdew JP, Burke K, Ernzerhof M (1996). Generalized gradient approximation made simple. Phys. Rev. Lett..

[39] Becke AD (1993). Density-functional thermochemistry. III. The role of exact exchange. J. Chem. Phys..

[40] Lin Y-S, Tsai C-W, Li G-D, Chai J-D (2012). Long-range corrected hybrid meta-generalized-gradient approximations with dispersion corrections. J. Chem. Phys..

[41] Lin Y-S, Li G-D, Mao S-P, Chai J-D (2013). Long-range corrected hybrid density functionals with improved dispersion corrections. J. Chem. Theory Comput..

[42] Wang C-W, Hui K, Chai J-D (2016). Short- and long-range corrected hybrid density functionals with the D3 dispersion corrections. J. Chem. Phys..

[43] Grimme S (2006). Semiempirical hybrid density functional with perturbative second-order correlation. J. Chem. Phys..

[44] Chai J-D, Head-Gordon M (2009). Long-range corrected double-hybrid density functionals. J. Chem. Phys..

[45] Chai J-D, Mao S-P (2012). Seeking for reliable double-hybrid density functionals without fitting parameters: the PBE0-2 functional. Chem. Phys. Lett..

[46] Hui K, Chai J-D (2016). SCAN-based hybrid and double-hybrid density functionals from models without fitted parameters. J. Chem. Phys..

[47] Cohen AJ, Mori-Sánchez P, Yang W (2012). Challenges for density functional theory. Chem. Rev..

[48] Gryn’ova G, Coote ML, Corminboeuf C (2015). Theory and practice of uncommon molecular electronic configurations. WIREs Comput. Mol. Sci..

[49] Chai J-D (2012). Density functional theory with fractional orbital occupations. J. Chem. Phys..

[50] Chai J-D (2014). Thermally-assisted-occupation density functional theory with generalized-gradient approximations. J. Chem. Phys..

[51] Chai J-D (2017). Role of exact exchange in thermally-assisted-occupation density functional theory: a proposal of new hybrid schemes. J. Chem. Phys..

[52] Wu C-S, Chai J-D (2015). Electronic properties of zigzag graphene nanoribbons studied by TAO-DFT. J. Chem. Theory Comput..

[53] Yeh C-N, Chai J-D (2016). Role of Kekulé and non-Kekulé structures in the radical character of alternant polycyclic aromatic hydrocarbons: a TAO-DFT study. Sci. Rep.

[54] Wu C-S, Lee P-Y, Chai J-D (2016). Electronic properties of cyclacenes from TAO-DFT. Sci. Rep..

[55] Tsivion E, Long JR, Head-Gordon M (2014). Hydrogen physisorption on metal-organic framework linkers and metalated linkers: a computational study of the factors that control binding strength. J. Am. Chem. Soc..

[56] Grimme S (2006). Semiempirical GGA-type density functional constructed with a long-range dispersion correction. J. Comput. Chem..

[57] Grimme S, Hansen A, Brandenburg JG, Bannwarth C (2016). Dispersion-corrected mean-field electronic structure methods. Chem. Rev..

[58] Shao Y (2015). Advances in molecular quantum chemistry contained in the Q-Chem 4 program package. Mol. Phys..

[59] Rivero P, Jiménez-Hoyos CA, Scuseria GE (2013). Entanglement and polyradical character of polycyclic aromatic hydrocarbons predicted by projected Hartree-Fock theory. J. Phys. Chem. B.

[60] Boys SF, Bernardi F (1970). The calculation of small molecular interactions by the differences of separate total energies. Some procedures with reduced errors. Mol. Phys..

[61] Löwdin P-O, Shull H (1956). Natural orbitals in the quantum theory of two-electron systems. Phys. Rev..

[62] Okamoto Y, Miyamoto Y (2001). Ab initio investigation of physisorption of molecular hydrogen on planar and curved graphenes. J. Phys. Chem. B.

[63] Breneman CM, Wiberg KB (1990). Determining atom-centered monopoles from molecular electrostatic potentials. The need for high sampling density in formamide conformational analysis. J. Comput. Chem..

[64] Durgun E, Ciraci S, Yildirim T (2008). Functionalization of carbon-based nanostructures with light transition-metal atoms for hydrogen storage. Phys. Rev. B.

[65] Chakraborty B, Modak P, Banerjee S (2012). Hydrogen storage in yttrium-decorated single walled carbon nanotube. J. Phys. Chem. C.

[66] Lemmon, E. W. In *Handbook of Chemistry and Physics* 96th edn (eds Haynes, W. M. *et al*.) Section 6, 21–37 (CRC Press, 2016).

[67] Li J (2003). Theoretical evaluation of hydrogen storage capacity in pure carbon nanostructures. J. Chem. Phys..

[68] Liu C-S, An H, Guo L-J, Zeng Z, Ju X (2011). Theoretical realization of cluster-assembled hydrogen storage materials based on terminated carbon atomic chains. J. Chem. Phys..

